# The smallest chimera state for coupled pendula

**DOI:** 10.1038/srep34329

**Published:** 2016-10-07

**Authors:** Jerzy Wojewoda, Krzysztof Czolczynski, Yuri Maistrenko, Tomasz Kapitaniak

**Affiliations:** 1 Division of Dynamics, Technical University of Lodz, Stefanowskiego 1/15, 90-924 Lodz, Poland; 2 Institute of Mathematics and Centre for Medical and Biotechnical Research, National Academy of Sciences of Ukraine, Tereshchenkivska st. 3, 01030 Kyiv, Ukraine; 3 Institut fur Theoretische Physik, Technische Universitat Berlin, Hardenbergstrasse 36, 10623 Berlin, Germany

## Abstract

Chimera states in the systems of coupled identical oscillators are spatiotemporal patterns in which different groups of oscillators can exhibit coexisting synchronous and incoherent behaviors despite homogeneous coupling. Although these states are typically observed in large ensembles of oscillators, recently it has been suggested that chimera states may occur in the systems with small numbers of oscillators. Here, considering three coupled pendula showing chaotic behavior, we find the pattern of the smallest chimera state, which is characterized by the coexistence of two synchronized and one incoherent oscillator. We show that this chimera state can be observed in simple experiments with mechanical oscillators, which are controlled by elementary dynamical equations derived from Newton’s laws. Our finding suggests that chimera states are observable in small networks relevant to various real-world systems.

The coexistence of the phase locked oscillators with desynchronized and incoherent oscillators in the network of identical oscillators creates the spatiotemporal patterns known as chimera states^1^,^2^,^3^,^4^,^5^,^6^,^7^,^8^,^9^,^10^,^11^,^12^,^13^,^14^,^15^,^16^,^17^,^18^,^19^,^20^,^21^,^22^,^23^. These patterns are typical for the large networks of different topologies and have been reported both in simulations^1^,^2^,^3^,^4^,^5^,^6^,^7^,^8^,^9^,^10^,^11^,^12^,^13^,^14^,^15^,^16^ and experiments^17^,^18^,^19^,^20^,^21^,^22^,^23^. Recently it has been suggested that chimera states can also be observed in small networks^24^,^25^,^26^. Ashwin & Burylko^24^ have defined a weak chimera state as one referring to a trajectory in which two or more oscillators are frequency synchronized and one or more oscillators drift in phase and frequency with respect to the synchronized group. First, it has been found out that these states can be observed in small networks of as few as 4 phase oscillators^24^,^25^,^26^.

Here, we show that the pattern of the smallest chimera state, which is characterized by two synchronized oscillators and one incoherent oscillator can be observed in the networks of 3 identical nodes. As the proof of the concept we use the network of coupled Huygens clocks^27^, i.e., the system of coupled pendula which are excited by the escapement clock’s mechanism^28^,^29^,^30^.

We consider the system of *3* coupled pendula shown in Fig. 1(a). which is shown in Fig. 1(a). Pendula of length *l*, mass *m* and moment of inertia *B* which hung from the unmovable disc are coupled to the nearest neighbor through the linear spring with stiffness coefficient *k*_x_ and linear dampers with damping coefficient *c*_x_ (shown in red). Pendula’s displacements are given by angles *ϕ*_*i*_. The springs and the dampers are connected to each pendulum at distance *l*_*s*_ from the pivot. Additionally, the motion of each pendulum is damped by the linear damper characterized by damping coefficient *c*_φ_. The energy is transmitted to each pendulum by the escapement mechanism which generate excitation torque *M*_D_ (in the first stage when 0 < *ϕ*_*i*_ < *γ*_*N*_ then *M*_*D*_ = *M*_*N*_ and when *ϕ*_*i*_ < 0 then *M*_*D*_ = 0 and for the second stage for −*γ*_*N*_ < *ϕ*_*i*_ < 0 *M*_*D*_ = −*M*_*N*_ and for *ϕ*_*i*_ > 0 *M*_*D*_ = 0)^28^,^29^. The described system can be experimentally implemented using three metronomes whose pendula are connected by the spring elements as shown in Fig. 1(b). The metronomes’ parameters, details about coupling and measurement techniques are given in the Methods. Each uncoupled pendulum is multistable and has three attractors: fixed points *A*_*1*_^*+*^, *A*_*1*_^*−*^ 
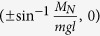
 and limit cycle *A*_*2*_ shown in Fig. 1(c). The boundaries between the basins of attraction of *A*_*1*_^*+*^ and *A*_*1*_^*−*^ attractors and *A*_*2*_ attractor are given by the ellipsoid 
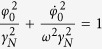
, where ω is frequency of oscillations and *γ*_*N*_ is constant given by the design of the escapement mechanism. The basins of attraction of *A*_*1*_^*+*^*, A*_*1*_^*−*^ and *A*_*2*_ are shown respectively in yellow and green colors. The boundaries between basins of *A*_*1*_^*+*^ and *A*_*1*_^*−*^ attractors have not been determined as they play no role in the explanation of the observed behavior.

The dynamics of the system in Fig. 1(a) can be analyzed using the equations of motion which are derived from the principles of classical mechanics (see the Methods). Due to the model of the escapement mechanism these equations are not differentiable so is the limit cycle *A*_*2*_ (discontinuity is shown in the inlet of Fig. 1(c)).

## Results

**Figure 1:**
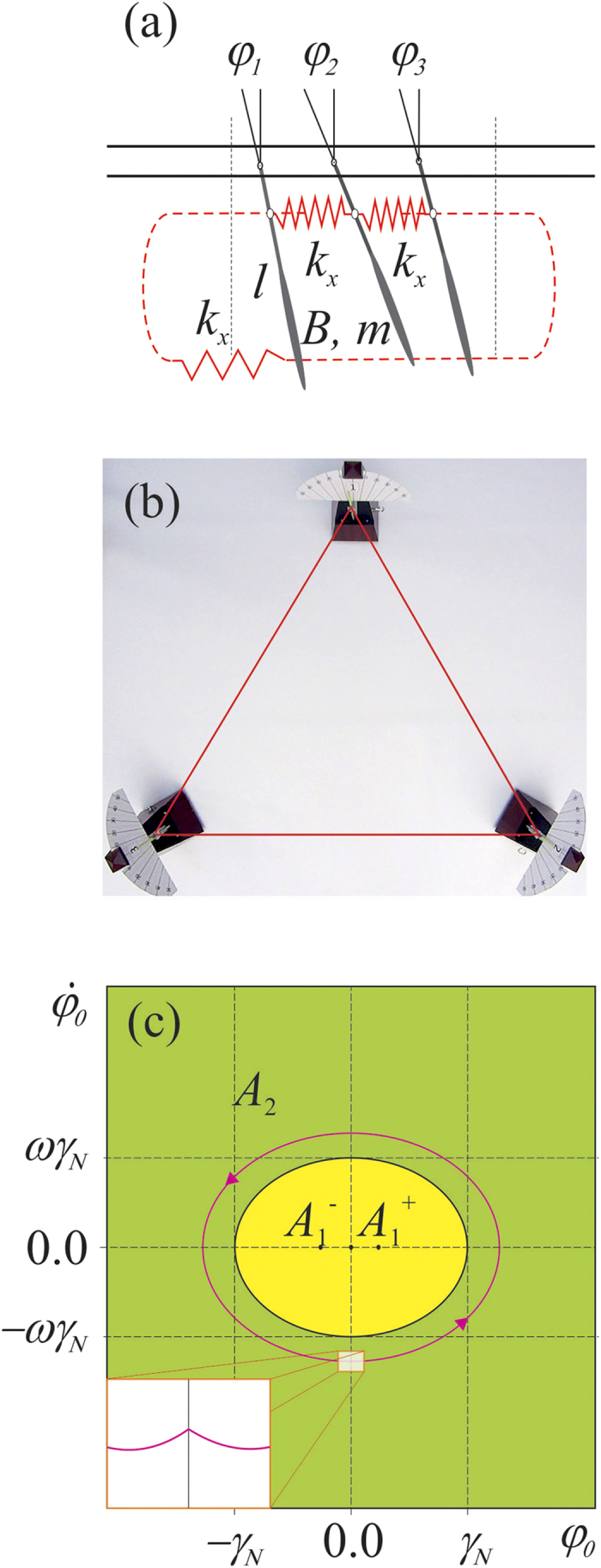
(**a**) 3 pendula coupled on the ring through springs and dampers, (**b**) experimental implementation of the system of Fig. 1(a) with *3* metronomes which pendula are coupled by spring elements, (**c**) coexisting attractors of each uncoupled pendulum (metronome).

**Figure 2:**
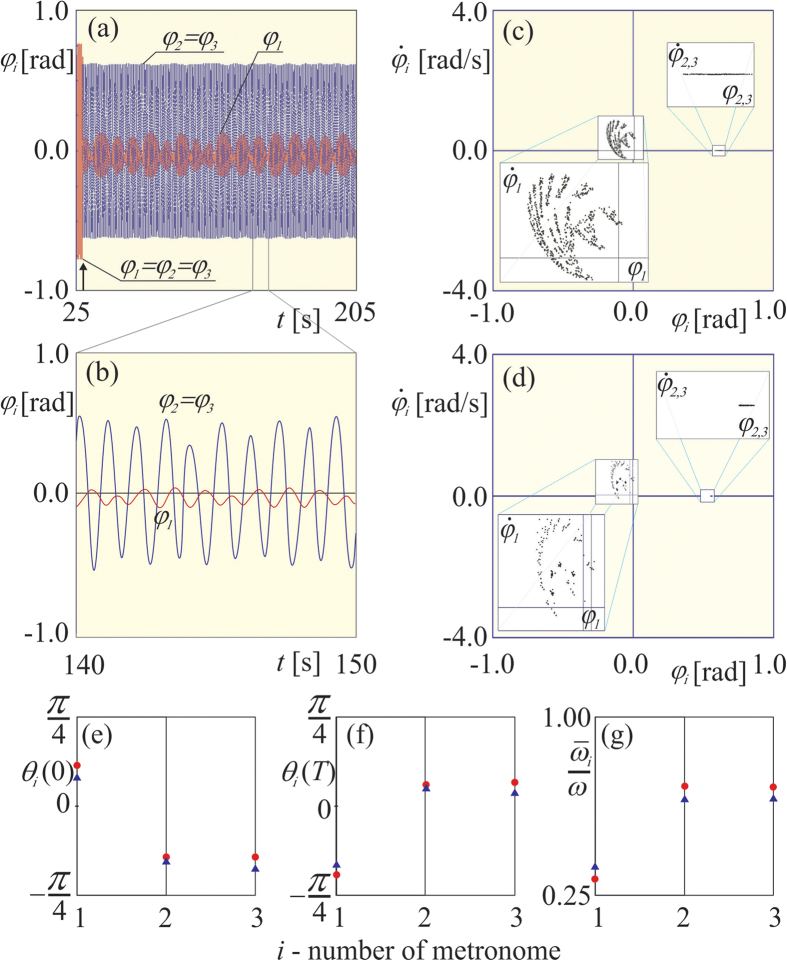
(**a**) Time series of displacement of all metronomes’ pendula *ϕ*_1−3_(*t*), originally all pendula have been synchronized, at the time indicated by arrow pendulum 1 has been stopped for a moment, (**b**) enlargement of the part of (**a**), (**c**,**d**) Poincare maps of pendula 1–3, (**c**) numerical results, (**d**) experimental results (**e**,**f**) snapshots of the phases of each metronomes *θ*_1−3_(*t*), (**e**) initial phases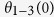, (f) final phases *θ*_1−3_(*T*), *T* = 1500[s], (**g**) mean frequencies of metronones 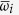
normalized by the frequency of uncoupled metronome ω (red dots and blue triangles indicate respectively numerical and experimental results).

For nonzero coupling stiffness *k*_*x*_ > 0 we observe broad range of parameters and initial conditions (whole basin of *A*_*2*_ attractor, i.e., green region in Fig. 1(c)) in which the synchronization of all metronomes emerges. We have assumed that all metronomes are synchronized when the phase differences between metronomes’ pendula are zero (in numerical simulations) or close to zero (in experiments), i.e., *ϕ*_*i*_(*t*) − *ϕ*_*j*_(*t*) = 0 (in numerical simulations) and *ϕ*_*i*_(*t*) − *ϕ*_*j*_(*t*) ≈ 0 (in experiments), *i, j* = *1, 2, 3, i* *≠* *j*. For sufficiently small coupling stiffness *k*_*x*_ (smaller than the threshold value *k*_*th*_) chimera states can be generated by the perturbation of the state of complete synchronization (one pendulum is stopped for a moment, i.e., when *ϕ*_*i*_ = 0, 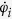 is set to 0) as can be seen in Fig. 2(a–d). Figure 2(a) presents time series of the displacement of all metronomes’ pendula *ϕ*_1−3_(*t*). The perturbation has been introduced to metronome 1 at the time indicated by the arrow. One can observe that pendula 2 and 3 are synchronized, the phase difference between them is equal to zero (*ϕ*_2_(*t*) − *ϕ*_3_(*t*) = 0) and pendulum 1 performs uncorrelated oscillations (*ϕ*_1_(*t*) − *ϕ*_2,3_(*t*) ≠ *constant*). Notice that the amplitudes of the oscillations are not equal and all pendula exhibit chaotic oscillations as can be seen in the enlargement shown in Fig. 2(b). The uncorrelated behavior of metronome 1 is confirmed in Poincare maps shown in Fig. 2(c,d). The points for the maps have been taken in the time moments when *ϕ*_2_(*t*) and *ϕ*_3_(*t*) reach maximum (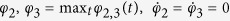). The numerical (Fig. 2(c)) and experimental (Fig. 2(d)) maps show that the behavior of synchronized metronomes 2 and 3 is restricted to the short interval on the line (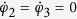) while 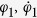 points of metronome 1 are distributed in the set bounded by white squares in the center of Fig. 2(d,c). To show that the observed behavior is a chimera state we calculated phases of each metronome *θ*_1−3_(*t*), (using the Fourier transformation - see Methods). Figure 2(e,f) shows that the phases of two metronomes (2 and 3) are approximately equal and different from the phase of metronome 1, i.e., *θ*_2_ ≈ *θ*_3_ ≠ *θ*_1_ (red dots and blue triangles indicate respectively numerical and experimental results). The mean frequencies of each metronome 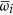 normalized by the frequency of uncoupled metronome ω are shown in Fig. 2(g). It is clearly visible that the drifting pendulum 1 is not frequency synchronized to the others. For the coupling stiffness *k*_*x*_ > *k*_*th*_ after the transient the perturbed metronome synchronizes with other two metronomes so the complete synchronization is restored (there are no phase differences between metronomes’ pendula).

Generally, in the described system the state of complete synchronization of all pendula (see Movie M1) co-exists with the state of partial phase synchronization in which two pendula oscillate in antiphase and the third one is at rest (see Movie M2) and the smallest chimera state (see Movie M3). Chimera state can be obtained also from random initial conditions when initial conditions of two metronomes belong to the basin of attractor *A*_*2*_ and initial conditions of the third one to the basins of attractors *A*_*1*_^*+*^ and *A*_*1*_^*−*^.

In the considered system of coupled metronomes chimera states can be observed due to: (i) the self-exited nature of its oscillations (ii) the multistability (co-existence of *A*_*1*_^*+*^*, A*_*1*_^*−*^ and *A*_*2*_ attractors) of each metronome, and (iii) sufficiently small (too small to forced synchronization via the energy transfers between metronomes^28^,^29^,^30^). These conditions can be generalized for the networks of coupled mechanical oscillators.

In summary, we have constructed the simple experimental setup to show the existence of the smallest chimera state in the network of three coupled pendula. The nodes in the network are locally coupled pendula (Huygens’ clocks realized by metronomes). We observe the formation of coexisting coherent (two synchronized pendula) and incoherent (the third pendulum) groups. This behavior is observed experimentally and confirmed in numerical simulations. It seems that such chimera states are common in the small networks of coupled multistable systems.

## Methods

The dynamics of the system of coupled pendula shown in Fig. 1(a) is given by:


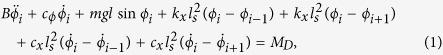


where *i* = 1, 2, 3, *φ*_0_ = *φ*_n_, *φ*_n+1_ = *φ*_1_. System (1) is symmetrical on the ring, i.e., pendulum *i* is coupled with pendula *i* + 1 and *i* − 1 (local coupling).

### Numerical simulations

The following parameter values have been used: *m* = 0.044 [kg], *l* = 0.011[m], *l*_*s*_ = 0.005[m], *B* = 0.0000974 [kgm^2^], *c*_φ_ = 0.00000107 [Nms], *M*_D_ = 0.00022 [Nm], γ_N_ = 17°, *c*_x _= 0.035 [Ns/m], *k*_x _= 0.444 [N/m]. The frequency of uncoupled metronome’s pendulum is equal to *ω *= 6.97[s^−1^]. With these parameters values the escapement mechanism generates oscillations of the uncoupled pendulum with amplitude *A* ≈ 0.75 [rad] ≈ 43°. The 4^th^ order Runge-Kutta method has been used for integration of eq. (1). The phases *θ*_1−3_(*t*) of the metronomes are obtained from numerical and experimental time series *ϕ*_1−3_(*t*) using Hilbert transformation: 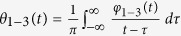. Further, the phases are used to obtain the averaged frequencies over time *T*: 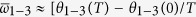. The averaging has been performed over the time interval *T *= 1500 [s].

### Experimental visualization

The set of 3 coupled metronomes shown in Fig. 1(b) has been used to confirm experimentally the existence of chimera states in the small network. Wittner Maelzel metronomes (Model No. 802K) covering frequency range of 40 (largo) to 208 (prestissimo) tics per minute, with a standard deviation of relative frequencies of ~1% have been used. Depending on the adjusted frequency each metronome ticks for a duration of approximately 25 min (when fully wound up). We have measured the angular displacements of metronomes’ pendula *φ*_i_ to quantitatively analyze the behavior of the coupled metronomes

The metronomes have been placed on the equilateral triangle as shown in Fig. 1(b). The triangle’s side has the length of 0.43 [m]. We have used the rubber bands (with the cross section radius 0.0001 [m]) of the length 0.43 [m] connecting the nearest neighbors as the spring elements. The estimated stiffness coefficient *k*_*x*_ is equal to 0.444 [N/m]. The threshold value on coupling stiffness is equal to *k*_*th*_ = 1.51[N/m]. The motion of the set of coupled metronomes has been recorded in two ways: (i) with a single Phantom v711 camera capable of high speed image acquisition, one at the time of a record (to obtain data shown in Fig. 2(c), and (ii) with the set of 4 general purpose devices: 3 Canon 5D facing directly each metronome’s pendulum respectively plus Sony HDR-MV1 observing the overall behavior from the top of the rig (Figs 1(b) and 2(d) and Movie W1). High speed camera Phantom v711 camera has been set to the speed of 100 or 150 fps recording in order to cover long time of the oscillator’s work, more than 1500 periods. The markers have been applied at the arms of the metronomes for further investigation with motion analysis program. TEMA software by Image Systems has been applied to consecutive movies (image sequences) which delivered digital values of angle, velocity and acceleration data obtained from tracing the markers on the arm of each metronome. Such time series allowed for the construction of the map presented in Fig. 2(c,d). As the method of observation, 4 recordings from 4 general purpose cameras have been gathered as the combination of views of behavior of the investigated oscillators in chosen examples. The final, rendered view from them has been synchronized within single frame accuracy.

## Additional Information

**How to cite this article**: Wojewoda, J. *et al*. The smallest chimera state for coupled pendula. *Sci. Rep.* **6**, 34329; doi: 10.1038/srep34329 (2016).

## Supplementary Material

Supplementary Information

Supplementary Information

Supplementary Information

Supplementary Information
