# Supplementary material for: The smallest chimera state for coupled pendula
Source: Sci Rep. 2016 Oct 7;6:34329. doi: 10.1038/srep34329 (PMC5054381; doi:10.1038/srep34329)
Supplement: Supplementary Information [file srep34329-s4.pdf]

The smallest chimera state for coupled pendula

Jerzy Wojewoda[1], Krzysztof Czolczynski[1], Yuri Maistrenko[1,2,3], & Tomasz Kapitaniak[1*]

[1]Division of Dynamics, Technical University of Lodz, Stefanowskiego 1/15, 90-924 Lodz, Poland,
[2]Institute of Mathematics and Centre for Medical and Biotechnical Research, National Academy of Sciences of Ukraine, Tereshchenkivska st. 3, 01030 Kyiv, Ukraine
[3]Institut fur Theoretische Physik, Technische Universitat Berlin, Hardenbergstrasse 36, 10623 Berlin, Germany

*corresponding author: tomaszka@p.lodz.pl

Supplementary information

Movie M1. The synchronous state of three metronomes.

Movie M2. The state in which two metronomes oscillate in antiphase and the third one is at rest.

Movie M3. The smallest chimera state.
